# Invasive Giant Goldenrod (*Solidago gigantea* Aiton): Phytochemical Profiling and Evaluation of Chemopreventive and Antimicrobial Activities

**DOI:** 10.3390/molecules31101552

**Published:** 2026-05-07

**Authors:** Elżbieta Gębarowska, Benita Wiatrak, Natalia Pachura-Hanusek, Karolina Budek, Martyna Gębarowska, Tomasz Gębarowski

**Affiliations:** 1 Wrocław University of Environmental and Life Sciences, Department of Plant Protection, Laboratory of Biogeochemistry and Environmental Microbiology, Grunwaldzka Str. 53, 50-375 Wroclaw, Poland; karolina.budek@upwr.edu.pl; 2 Wrocław Medical University, Department of Pharmacology, J. Mikulicza-Radeckiego Str. 2, 50-345 Wroclaw, Poland; benita.wiatrak@umw.edu.pl; 3 Wrocław University of Environmental and Life Sciences, Department of Environment Hygiene and Animal Welfare, Chełmońskiego Str. 38c, 50-375 Wroclaw, Poland; natalia.pachura@upwr.edu.pl; 4 Wrocław University of Environmental and Life Sciences, Department of Biostructure and Animal Physiology, Veterinary Biotechnology Student Research Club “Refectio”, Kożuchowska Str. 1, 51-631 Wroclaw, Poland; 5 Wrocław University of Environmental and Life Sciences, Department of Biostructure and Animal Physiology, Kożuchowska Str. 1, 51-631 Wroclaw, Poland; tomasz.gebarowski@upwr.edu.pl

**Keywords:** *Solidago gigantea*, invasive plant, phytochemistry, polyphenols, triterpenoids, antimicrobial activity, chemoprevention, ROS modulation, P-glycoprotein, LC-MS/MS, GC-MS

## Abstract

*Solidago gigantea* Aiton is an invasive plant species rich in bioactive secondary metabolites. The aim of this study was to characterize the phytochemical profile of an ethanolic *S. gigantea* extract and to evaluate its antibacterial and chemoprotective potential using in vitro models. Chemical analysis revealed a high content of phenolic compounds, dominated by chlorogenic acid, along with other phenolic acids and flavonoids, including rutin and quercitrin. The extract also contained saponins and a diverse lipophilic fraction composed of long-chain hydrocarbons, alcohols, fatty acids, phytosterols, and triterpenoids. The extract exhibited strong antibacterial activity against Gram-positive bacteria, including methicillin-resistant *Staphylococcus* strains, while Gram-negative bacteria and yeasts were less susceptible. In cancer cell models, the extract showed selective antiproliferative and cytotoxic effects, particularly in colorectal and breast cancer cell lines, including doxorubicin-resistant phenotypes, with minimal effects on normal fibroblasts. The extract also showed antioxidant and cytoprotective properties, reflected by a reduction in intracellular reactive oxygen species under both basal and oxidative stress conditions. Increased accumulation of rhodamine 123 in resistant cancer cells suggested a potential inhibition of P-glycoprotein-mediated efflux. Overall, the results indicate that *S. gigantea* extract exhibits multi-target biological activity associated with its polyphenolic composition, supporting its potential application in chemoprevention, adjuvant cancer therapy, and the control of Gram-positive bacterial infections.

## 1. Introduction

The rapid increase in antimicrobial resistance (AMR) and the growing global burden of cancer represent two of the most pressing challenges in modern medicine. The widespread and often inappropriate use of antibiotics has accelerated the emergence of multidrug-resistant (MDR) pathogens, significantly limiting the effectiveness of conventional therapies [1,2,3,4]. Similarly, the clinical efficacy of many anticancer drugs is compromised by toxicity and the development of resistance mechanisms, including drug efflux mediated by ATP-binding cassette (ABC) transporters such as P-glycoprotein (P-gp) [5,6,7,8]. These challenges have intensified interest in natural products as alternative or complementary sources of bioactive compounds.

Plant-derived secondary metabolites, particularly polyphenols such as flavonoids and phenolic acids, are widely recognized for their broad spectrum of biological activities. These compounds have been shown to exhibit antibacterial effects, especially against Gram-positive bacteria, including methicillin-resistant *Staphylococcus aureus* (MRSA), through mechanisms involving membrane disruption and interference with cellular processes [9,10]. In addition, polyphenols demonstrate antiproliferative and cytotoxic effects in various cancer cell models and may modulate multidrug resistance mechanisms, including the inhibition of ABC transporters [5,6,7,8].

Beyond their antimicrobial and anticancer properties, polyphenolic compounds play an important role in the regulation of cellular redox homeostasis. Reactive oxygen species (ROS) are involved in multiple physiological and pathological processes, including cell signaling, proliferation, and apoptosis. Dysregulation of redox balance contributes to cancer progression and cellular damage, whereas modulation of ROS levels represents a key mechanism of action for many natural compounds [7,11].

In vitro cell culture models provide a valuable tool for the preliminary evaluation of the biological activity and selectivity of natural products. Cell viability and proliferation are commonly assessed using complementary assays such as MTT and sulforhodamine B (SRB), which measure metabolic activity and total cellular protein content, respectively. In addition, intracellular ROS measurements enable the assessment of redox-related effects, while functional evaluation of multidrug resistance is frequently performed using rhodamine 123, a fluorescent substrate of P-glycoprotein [5,8].

*Solidago gigantea* Aiton (giant goldenrod) is a highly invasive plant species widely distributed across Europe, where it negatively affects native ecosystems [12]. Despite its invasive ecological impact, it represents a promising source of bioactive secondary metabolites. Previous studies have shown that species of the genus *Solidago* are rich in phenolic compounds, including chlorogenic acid and flavonoids such as rutin and quercetin derivatives, which are associated with antioxidant, antimicrobial, and anticancer activities [6,7,13].

Extracts from *Solidago* species have demonstrated selective antibacterial activity against Gram-positive bacteria, including MRSA strains, while Gram-negative bacteria and yeasts are generally less susceptible [13,14]. Moreover, cytotoxic and antiproliferative effects have been reported in various cancer cell models [8,14]. However, most studies have focused either on phytochemical characterization or on selected biological activities.

To date, integrated studies linking detailed phytochemical profiling with simultaneous evaluation of antimicrobial activity, cytotoxicity, redox modulation, and multidrug resistance mechanisms remain limited, particularly for *S. gigantea*. In addition, the contribution of lipophilic constituents to the biological activity of *Solidago* extracts has been insufficiently explored.

Therefore, the aim of the present study was to investigate the phytochemical composition and biological activity of *Solidago gigantea* extracts using an integrated in vitro approach. The extract was characterized using LC-MS/MS and GC-MS analyses. Antimicrobial activity was evaluated against a panel of bacterial (including methicillin-resistant isolates) and yeast strains. Cytotoxic and antiproliferative effects were assessed in normal and cancer cell lines, including drug-resistant models. In addition, oxidative stress and efflux transporter activity were analyzed to provide a comprehensive evaluation of the extract’s biological potential.

## 2. Results

### 2.1. Chemical Profiling

Plant extracts usually constitute complex mixtures of multiple chemical fractions. In this study, an integrated approach combining phytochemical profiling of both polar and non-polar fractions with biological activity evaluation was applied to achieve a comprehensive characterization of the extract.

The results of the chemical profile analysis are presented in Table 1. LC-MS/MS analysis showed that the ethanolic extract of *Solidago gigantea* is characterized by a polyphenolic profile dominated by phenolic acids and flavonol glycosides. Quantitatively, phenolic acids accounted for approximately 57.6% of the total identified compounds, while flavonoids represented 42.4%. Among all quantified compounds, chlorogenic acid was the most abundant constituent, followed by rutin and quercitrin. Other major compounds included ferulic and protocatechuic acids, as well as quercetin. Lower concentrations were observed for caffeic, syringic, and *p*-coumaric acids, together with neohesperidin and kaempferol, while vanillic acid, taxifolin, polydatin, and coniferyl alcohol were detected only in trace amounts.

Within individual groups, chlorogenic acid constituted nearly 58% of the phenolic acid fraction, whereas rutin accounted for approximately 55% of the flavonoid fraction, confirming their dominant contribution to the extract composition.

Overall, the results indicate that the analyzed extract is rich in hydroxycinnamic acid derivatives and flavonol glycosides, with chlorogenic acid and rutin as the predominant constituents. The total content of identified phenolic compounds reached 234.27 TAE/g.

The investigated ethanolic extract fraction contained 64.2 mg AE/g of total saponins, as determined by spectrophotometry.

GC-MS analysis of the *Solidago gigantea* extract after BSTFA derivatization (Table 2 and Figure S2) revealed a chemically diverse lipophilic fraction composed mainly of long-chain hydrocarbons, fatty acids, aliphatic alcohols, sterols, and pentacyclic triterpenoids.

Quantitatively, the extract was dominated by long-chain hydrocarbons (~43%) and aliphatic alcohols (~28%), followed by triterpenoids (~19%), while fatty acids and other minor constituents accounted for the remaining fraction.

Among individual compounds, *n*-hentriacontane (21.37 mg/g) and *n*-nonacosane (19.18 mg/g) were the most abundant hydrocarbons, confirming the major contribution of cuticular wax-derived constituents. High levels of long-chain alcohols were also detected, particularly 1-hexacosanol (16.81 mg/g) and 1-triacontanol (10.58 mg/g).

The triterpenoid fraction was mainly represented by erythrodiol (15.55 mg/g) and uvaol (9.01 mg/g), accompanied by α- and β-amyrin and other oleanane- and lupane-type derivatives. Fatty acids, including palmitic (6.39 mg/g), linoleic (4.15 mg/g), and α-linolenic acids (3.60 mg/g), were present in moderate amounts.

Overall, these results indicate a substantial contribution of lipophilic constituents associated with plant surface waxes, complementing the polyphenolic fraction identified by LC-MS/MS and highlighting the dual chemical nature of the extract.

The chromatographic profile additionally confirmed the presence of saturated and unsaturated fatty acids (as trimethylsilyl derivatives), as well as minor constituents such as phytol, stigmasterol, and triacontanol, together with a range of pentacyclic triterpenoids.

Quantified relative to the internal standard, the total amount of volatile organic compounds (VOCs) in the investigated fraction was 8 mg/g. GC-MS analysis of the *Solidago gigantea* distillate identified 38 compounds, accounting for 98.99% of the total composition (Table 3 and Figure S1). The chemical profile was dominated by oxygenated sesquiterpenes (48.54%), followed by monoterpene hydrocarbons (16.20%) and oxygenated monoterpenes (15.49%).

Among the identified constituents, the most abundant compounds were caryophyllene oxide (15.68%), humulene epoxide II (14.19%), and spathulenol (9.69%), indicating a predominance of oxygenated sesquiterpene derivatives. Monoterpenes such as α-pinene (9.12%) and β-pinene (2.78%) were also present in notable amounts.

Overall, the volatile profile of *S. gigantea* is characterized by a high proportion of oxygenated terpenoids, which may contribute to its biological activity and complement the non-volatile fractions identified by GC-MS after BSTFA derivatization.

### 2.2. Microbial Assay

The antibacterial activity of the *Solidago gigantea* extract was preliminarily evaluated using the agar disk diffusion method. The results were expressed as inhibition zone diameters (mm) and as the inhibition coefficient (I) relative to the positive control (Table 4). The extract exhibited differential antibacterial activity depending on the tested microorganism.

The strongest inhibitory effects were observed against Gram-positive bacteria. The largest inhibition zone was recorded for *Staphylococcus epidermidis* PCM 2118 (22.4 ± 0.33 mm), corresponding to 70.4% of the activity of the positive control. Moderate antibacterial activity was observed against other staphylococcal strains, including *Staphylococcus aureus* PCM 2054 and PCM 458, with inhibition zones of 13.0 ± 0.33 mm (48.8%) and 10.7 ± 0.38 mm (39.5%), respectively.

Methicillin-resistant strains (*S. aureus* MRSA ATCC 33592 and ATCC 3144, as well as *S. epidermidis* MRSE PCM 2532) exhibited inhibition zones comparable to those of the positive control, with no statistically significant differences (*p* > 0.05). In contrast, the Gram-negative bacterium *Escherichia coli* PCM 2057 showed low susceptibility to the extract, while no inhibitory effect was observed against *Candida albicans* PCM 2566.

The analysis of the bacteriostatic and fungicidal activity of *S. gigantea* extract varied depending on the tested microorganism (Table 5). The extract exhibited activity primarily against Gram-positive bacteria, whereas Gram-negative bacteria and yeasts showed no growth inhibition within the tested concentration range (MIC and MBC > 10 mg/mL). For Gram-positive bacteria, 50% growth inhibition (MIC_50_) ranged from 0.9 to 5.1 mg/mL, while 90% growth inhibition (MIC_90_) ranged from 3.9 to 9.6 mg/mL. The lowest MIC values were observed for *S. aureus* PCM 2054 and *B. cereus* PCM 2019, including some methicillin-resistant strains (MRSA and MRSE), which required slightly higher extract concentrations, reflecting their reduced susceptibility.

**Figure 1:**
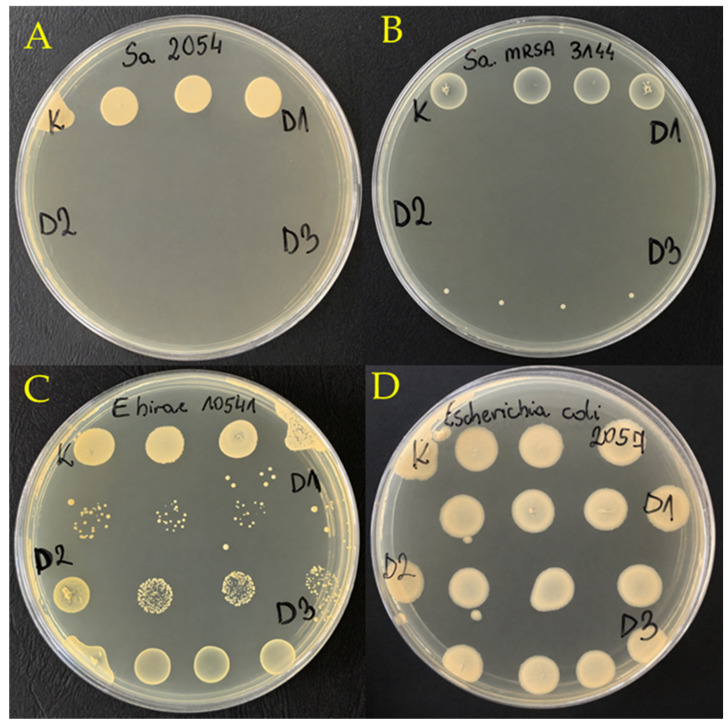
Representative bactericidal or growth-reducing effect of *S. gigantea* extract on bacterial colonies: (**A**) *S. aureus* PCM 2054; (**B**) *S. aureus* MRSA PCM 3144; (**C**) *Enterococcus hirae* PCM 10541; (**D**) *E. coli* PCM 2057 (no reduction in colony growth observed). Treatments: K—control, D1—10 mg/mL, D2—5 mg/mL, D3—2.5 mg/mL.

The bactericidal and growth-reducing effects of the *S. gigantea* extract on selected bacterial strains are illustrated in Figure 1. Representative colonies of *S. aureus* PCM 2054, MRSA PCM 3144, *E. hirae* PCM 10541, and *E. coli* PCM 2057 demonstrate the concentration-dependent reduction in colony size and viability, with no observable effect on *E. coli* at the tested concentrations.

### 2.3. Cytotoxic Activity of Solidago gigantea Extract (MTT Assay)

Cytotoxic activity of the ethanolic extract of *Solidago gigantea* was evaluated using the MTT assay in a panel of cell lines comprising normal human dermal fibroblasts (NHDF) and cancer cell lines with different sensitivity profiles and resistance phenotypes, including breast cancer (MCF-7, MCF-7/DX), colorectal cancer (LoVo, LoVo/DX), lung carcinoma (A549), and monocytic leukemia (THP-1). Cells were exposed to the extract at concentrations ranging from 12.5 to 5000 µg/mL for 24 h. The half-maximal inhibitory concentration (IC_50_) values were calculated by nonlinear regression using a four-parameter logistic model based on five independent biological replicates (Table 6).

The *S. gigantea* extract exhibited a clear, cell-type-dependent cytotoxic effect (Table 6). The highest sensitivity was observed in colorectal cancer cell lines, particularly in the doxorubicin-resistant LoVo/DX cells, which showed the lowest IC_50_ value (50.7 ± 7.8 µg/mL). A similarly strong response was noted for the parental LoVo line (IC_50_ = 98.9 ± 11.2 µg/mL), as well as for MCF-7 breast cancer cells (IC_50_ = 78.2 ± 9.4 µg/mL), indicating pronounced susceptibility of these tumor models to the extract.

In contrast, the doxorubicin-resistant MCF-7/DX cells displayed a significantly higher IC_50_ value (362.1 ± 38.5 µg/mL) compared to the parental MCF-7 line (*p* < 0.01), suggesting partial cross-resistance. No statistically significant difference was observed between LoVo and LoVo/DX cells (*p* > 0.05), indicating that resistance mechanisms associated with doxorubicin do not reduce the cytotoxic efficacy of the *S. gigantea* extract in this colorectal cancer model.

Moderate sensitivity was detected in A549 lung carcinoma cells (IC_50_ = 274.6 ± 31.7 µg/mL), whereas THP-1 monocytic cells were markedly less sensitive, with an IC_50_ of 1862.3 ± 210.5 µg/mL. The lowest cytotoxicity was observed in normal NHDFs. Since cell viability remained above 69% at the highest tested concentration (5000 µg/mL), the IC_50_ value for NHDF was estimated by extrapolation of the sigmoidal dose–response curve and reached approximately 7180 ± 580 µg/mL, confirming very low toxicity of the extract toward normal cells.

Selectivity indices (SI) were calculated to assess therapeutic relevance (Table 7). High SI values observed for LoVo/DX, LoVo, and MCF-7 indicate pronounced selectivity toward cancer cells relative to normal fibroblasts.

### 2.4. Influence of Solidago gigantea Extract on Cell Proliferation (SRB Assay)

The antiproliferative activity of the *S. gigantea* extract was evaluated using the SRB assay in normal human dermal fibroblasts (NHDF) and cancer cell lines (A549, THP-1, LoVo, LoVo/DX, MCF-7, and MCF-7/DX).

**Figure 2:**
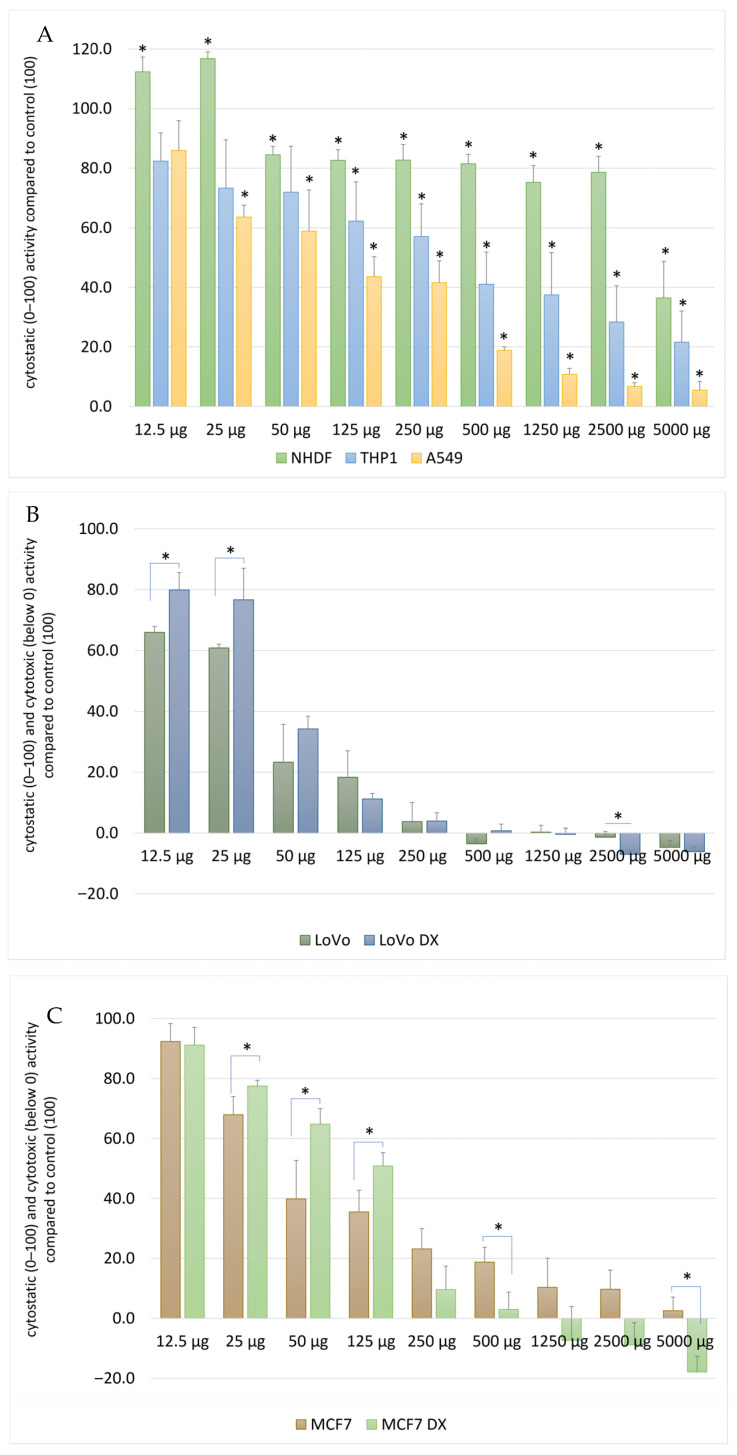
Antiproliferative effect of *Solidago gigantea* extract assessed by the SRB assay. Cell proliferation was evaluated after 24 h exposure to *S. gigantea* extract at concentrations ranging from 12.5 to 5000 µg/mL in (**A**) normal human dermal fibroblasts (NHDF), (**B**) cancer cell lines A549 and THP-1, and (**C**) colorectal and breast cancer cell lines (LoVo, LoVo/DX, MCF-7, MCF-7/DX). Results are expressed as percentage of cell growth relative to untreated control cells (* *p* < 0.05). Values below the time-zero level indicate cytotoxic effects. Data represent mean values ± SD from independent experiments.

In NHDF cells, the extract exhibited only a weak cytostatic effect, which was observed mainly at the highest concentrations (≥1250 µg/mL), with no significant cytotoxicity. In contrast, cancer cells showed markedly higher sensitivity to the extract (Figure 2A).

In A549 and THP-1 cell lines, a gradual, dose-dependent inhibition of cell proliferation was observed, reaching values below 50% of the control at concentrations ≥ 500 µg/mL. The strongest antiproliferative effect was recorded in colorectal cancer cell lines (LoVo and LoVo/DX) and breast cancer cell lines (MCF-7 and MCF-7/DX), where at the highest extract concentrations a cytotoxic effect was observed, reflected by SRB values below the time-zero level (Figure 2B,C).

Importantly, doxorubicin-resistant cell lines (LoVo/DX and MCF-7/DX) exhibited sensitivity comparable to, or only slightly lower than, their parental counterparts, indicating that the *S. gigantea* extract retains antiproliferative activity also against chemotherapy-resistant phenotypes.

### 2.5. Effect of Solidago gigantea Extract on Intracellular ROS Levels (DCF-DA Assay)

The level of intracellular reactive oxygen species (ROS) was evaluated using the DCF-DA assay in normal cell lines (NHDF and V79) and cancer cell lines (A549 and THP-1) after 24 h of incubation with *Solidago gigantea* extract at concentrations ranging from 12.5 to 5000 µg/mL.

**Figure 3:**
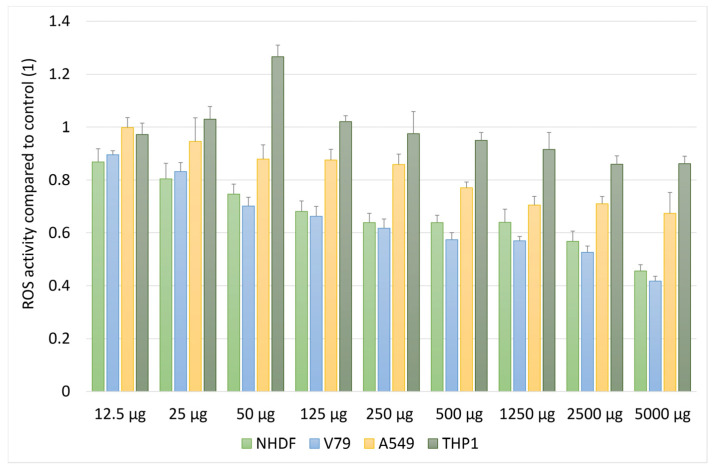
Effect of *Solidago gigantea* extract on intracellular ROS levels under basal conditions. Intracellular reactive oxygen species (ROS) levels were measured using the DCF-DA assay in normal (NHDF, V79) and cancer (A549, THP-1) cell lines after 24 h incubation with *S. gigantea* extract at concentrations ranging from 12.5 to 5000 µg/mL. Results are expressed as relative ROS levels compared to untreated control cells. Data represent mean values ± SD from independent experiments.

Under basal conditions (without induction of oxidative stress), the extract caused a dose-dependent reduction in ROS levels in all tested cell lines (Figure 3). This effect was particularly pronounced in NHDF and V79 cells, where at the highest concentrations (2500–5000 µg/mL), ROS levels decreased to approximately 40–50% of control values. A similar trend was observed in the cancer cell lines A549 and THP-1; however, the extent of ROS reduction was moderately lower, especially at lower concentration ranges.

**Figure 4:**
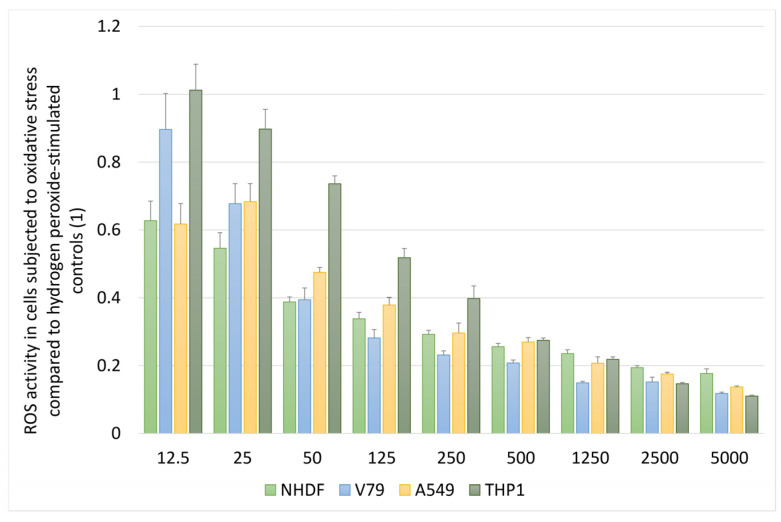
Protective effect of *Solidago gigantea* extract against hydrogen peroxide–induced oxidative stress. Cells (NHDF, V79, A549, and THP-1) were exposed to hydrogen peroxide (H_2_O_2_) to induce oxidative stress and subsequently treated with *S. gigantea* extract (12.5–5000 µg/mL). Intracellular ROS levels were assessed using the DCF-DA assay. Results are expressed relative to H_2_O_2_-stimulated control cells. Data represent mean values ± SD from independent experiments.

Under oxidative stress conditions induced by hydrogen peroxide (H_2_O_2_), the *S. gigantea* extract exhibited a strong protective effect, leading to a significant decrease in ROS levels compared to the H_2_O_2_-stimulated control. A marked reduction in DCF fluorescence was already observed at concentrations of 50–125 µg/mL, while at the highest doses ROS levels were reduced to approximately 10–20% of the oxidative stress control, regardless of the cell type (Figure 4).

### 2.6. Effect of Solidago gigantea Extract on Rhodamine 123 Accumulation

The activity of efflux transporters associated with multidrug resistance was assessed based on the intracellular accumulation of rhodamine 123 in the MCF-7/MCF-7-DX and LoVo/LoVo-DX cell line pairs.

**Figure 5:**
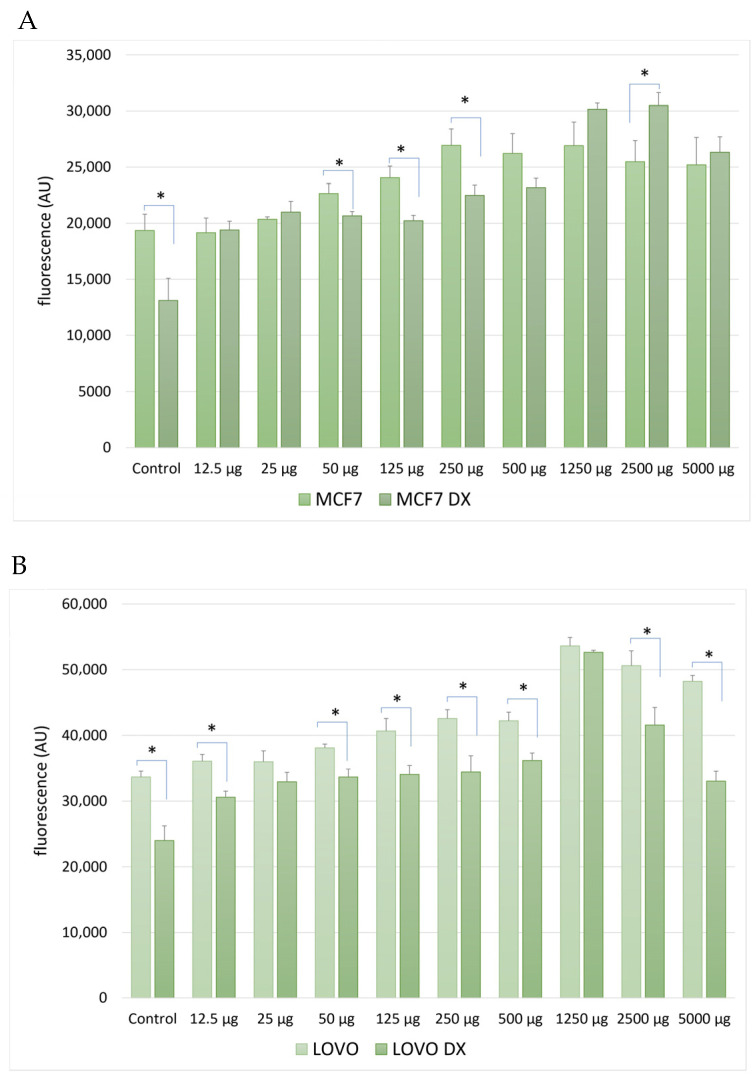
Effect of *Solidago gigantea* extract on rhodamine 123 accumulation in drug-sensitive and drug-resistant cancer cell lines. Intracellular accumulation of rhodamine 123 was evaluated in (**A**) MCF-7 and MCF-7/DX breast cancer cells and (**B**) LoVo and LoVo/DX colorectal cancer cells after 24 h incubation with *S. gigantea* extract at the indicated concentrations. Fluorescence intensity is expressed relative to untreated control cells (* *p* < 0.05). Increased rhodamine 123 accumulation in resistant cell lines indicates inhibition of P-glycoprotein (P-gp)–mediated efflux. Data represent mean values ± SD from independent experiments.

In drug-sensitive cells (MCF-7 and LoVo), rhodamine 123 fluorescence levels remained relatively stable, with only minor changes observed at the highest extract concentrations. In contrast, in the resistant cell lines (MCF-7/DX and LoVo/DX), the *S. gigantea* extract induced a significant, dose-dependent increase in intracellular rhodamine 123 accumulation, particularly at concentrations ≥ 125 µg/mL (Figure 5A,B).

The increased fluorescence indicates inhibition of P-glycoprotein (P-gp) activity, which is responsible for the active efflux of rhodamine 123 from resistant cells. This effect was more pronounced in LoVo/DX cells than in MCF-7/DX cells, suggesting differences in the modulation of multidrug resistance (MDR) mechanisms between the cancer cell lines.

## 3. Discussion

Building on our previous reports describing the antimicrobial potential of *Solidago gigantea* extracts against plant pathogens [15], the present study extends these findings by providing an integrated evaluation of the phytochemical composition and multi-target biological activity of *Solidago gigantea* extract in both microbial and mammalian cell models. As an invasive species, *S. gigantea* represents an abundant and underexplored source of bioactive compounds, with potential biomedical relevance.

### 3.1. Chemical Profile of the Solidago gigantea Extract

The ethanolic extract of *S. gigantea* was characterized by a complex, multi-fractional composition comprising a polar fraction (primarily polyphenols), a lipophilic fraction (long-chain hydrocarbons, fatty acids, and triterpenoids), and a volatile fraction (terpenoids). The polyphenolic fraction was dominated by chlorogenic acid and flavonoids such as rutin and quercetin, which are widely recognized for their antioxidant, anti-inflammatory, anticancer and antimicrobial properties [6,7,13,16,17].

The lipophilic fraction consisted mainly of aliphatic hydrocarbons, long-chain alcohols, and pentacyclic triterpenoids (e.g., erythrodiol and uvaol), which may contribute to membrane interactions and biological activity [18,19].

The volatile fraction (VOCs) was dominated by oxygenated sesquiterpenes, including caryophyllene oxide and humulene epoxide II, compounds previously associated with antimicrobial and anti-inflammatory effects [18,20].

Overall, the coexistence of polar and non-polar constituents suggests a potential synergistic contribution to the observed biological effects, supporting the multi-target activity of the extract [16,21].

### 3.2. Modulation of Oxidative Stress and Redox Homeostasis

The extract significantly reduced intracellular ROS levels under both basal and oxidative stress conditions in normal and cancer cell lines. This effect was particularly pronounced in non-transformed NHDFs at higher concentrations.

The observed antioxidant activity may be attributed to the presence of polyphenolic compounds, which are known to act as direct radical scavengers and modulators of cellular redox homeostasis [11,22,23]. In addition to direct ROS neutralization, polyphenols may enhance endogenous antioxidant defense systems, including the upregulation of antioxidant enzymes and modulation of redox-sensitive signaling pathways [11,22,23,24].

These findings are consistent with literature reports indicating that plant-derived phenolic compounds can effectively reduce oxidative stress and protect normal cells from ROS-induced damage in fibroblast models and other systems [24,25]. The stronger effect observed in non-transformed cells may reflect differences in basal redox status and antioxidant capacity between normal and cancer cells.

### 3.3. Antiproliferative and Cytotoxic Effects

The extract exhibited selective cytotoxic and antiproliferative activity toward cancer cells, particularly in colorectal and breast cancer models, while showing minimal toxicity toward normal fibroblasts. This selectivity was further supported by high selectivity index (SI) values.

The combined use of MTT and SRB assays enabled differentiation between metabolic activity (cell viability) and total protein content (cell biomass), providing complementary insight into cytotoxic and cytostatic effects [26,27]. The results indicate that the extract exerts both antiproliferative and cytotoxic effects, depending on the cell type and concentration.

Interestingly, drug-resistant cell lines (LoVo/DX and MCF-7/DX) retained sensitivity to the extract, suggesting that its mechanism of action may differ from that of conventional chemotherapeutic agents and may partially overcome resistance-associated pathways [6,7].

### 3.4. Modulation of Multidrug Resistance Mechanisms

The rhodamine 123 accumulation assay demonstrated increased intracellular fluorescence in resistant cancer cell lines following treatment with the extract, suggesting a potential inhibition of P-glycoprotein (P-gp)-mediated efflux activity. This observation is consistent with previous reports indicating that flavonoids and other polyphenols can modulate ABC transporter function [5,28].

However, these results should be interpreted with caution due to the lack of a specific positive control for P-gp inhibition and the absence of direct verification of P-gp expression levels in the tested cell lines.

Nevertheless, the MCF-7/DX and LoVo/DX models used in this study are well-established and widely reported to exhibit P-gp overexpression, as demonstrated in previous studies on multidrug resistance and ABC transporter activity [5]. It should be noted that doxorubicin-resistant sublines are commonly maintained under selective pressure, which is known to induce and stabilize P-gp overexpression.

### 3.5. Antimicrobial Activity

The extract demonstrated strong antibacterial activity against Gram-positive bacteria, including methicillin-resistant strains (MRSA and MRSE), while Gram-negative bacteria and yeasts were markedly less susceptible. This selectivity is consistent with known differences in cell envelope structure between Gram-positive and Gram-negative microorganisms [29,30,31].

The observed antimicrobial activity may be associated with polyphenolic compounds, which are known to disrupt bacterial membranes, interfere with enzymatic systems, and affect cellular metabolism [17,30]. Additionally, terpenoid components present in the volatile fraction may contribute to antimicrobial effects through membrane perturbation and increased permeability [17,19].

### 3.6. Limitations and Future Perspectives

Despite the comprehensive nature of the study, several limitations should be acknowledged. The lack of direct verification of P-gp expression and the absence of a specific positive control in the rhodamine 123 assay limit the mechanistic interpretation of multidrug resistance modulation. In addition, the IC_50_ value for NHDF cells was estimated by extrapolation beyond the tested concentration range and should therefore be interpreted with caution.

Future studies should focus on detailed molecular mechanisms, including transporter expression analysis, signaling pathway modulation, and in vivo validation of biological activity.

The *Solidago gigantea* extract exhibits multi-target biological activity, including antioxidant, antiproliferative, multidrug resistance-modulating, and selective antibacterial effects. These activities are likely associated with its complex phytochemical composition, particularly its high polyphenolic content. The findings support its potential application in chemoprevention, adjuvant cancer therapy, and the control of Gram-positive bacterial infections, although further studies are required to confirm its mechanisms of action and therapeutic relevance.

## 4. Materials and Methods

### 4.1. Plant Material and Extract Preparation

The plant material of *Solidago gigantea* Aiton was collected in September 2024, at the flowering stage, in the municipality of Wisznia Mała, Poland (51.213353, 17.046135). Prior to drying, the plant material was cut into sections of 20 ± 2 mm. The aboveground parts were dried in a drying cabinet at 25–30 °C for 72 h. The dried material (100 g, moisture content < 10%) was ground using a grinder (IKA M20, IKA-Werke, Staufen im Breisgau, Germany) and extracted three times with absolute ethanol at a 1:5 (*w*/*v*) ratio. The resulting mixture was shaken for 60 min on a rotary shaker (125 rpm), filtered, and concentrated using a rotary evaporator (VWR IKA, Radnor, PA, USA). The dried extracts were weighed (8.3 g) and stored at 4 °C until further analysis. A voucher specimen was deposited in the Laboratory of Biogeochemistry and Environmental Microbiology of Wrocław University of Environmental and Life Sciences under number 006/09/2024.

### 4.2. Chemical Analysis

#### 4.2.1. LC-MS Analysis

To identify phenolic acids and flavonoids, the crude ethanolic extract of *S. gigantea* was prepared from powdered aerial parts.

The dried extract was re-dissolved in ethanol prior to LC-MS analysis and filtered through a 0.22 µm syringe filter.

The analyses were performed in triplicate using an LCMS-8045 system (Shimadzu, Kyoto, Japan) equipped with an electrospray ionization source. Chromatographic separation was achieved on a Kinetex C18 column (2.6 µm, 100 Å, 100 × 3.0 mm; Phenomenex, Torrance, CA, USA). The mobile phase consisted of 0.1% (*v*/*v*) formic acid in water (A) and ethanol (B). The gradient elution started at 10% B, increased to 20% B within 5 min, reached 60% B at 10 min, and returned to initial conditions between 10 and 13 min, followed by 4 min equilibration. The flow rate was 0.30 mL/min, injection volume 5 µL, and column temperature 40 °C. Phenolic acids and flavonoids were identified using multiple reaction monitoring (MRM) mode (Table 8). Nitrogen was used as the nebulizing and drying gas. Analytical standards of phenolic acids and flavonoids, as well as LC–MS grade solvents (methanol, water, and formic acid), were purchased from Sigma-Aldrich (St. Louis, MO, USA).

Quantification was performed using external calibration curves prepared from analytical standards. Linearity was confirmed within the tested concentration ranges (R^2^ > 0.99). Limits of detection (LOD) and quantification (LOQ) were estimated based on signal-to-noise ratios of 3 and 10, respectively. All analyses were performed in triplicate, and the results are expressed as mean values ± standard deviation (SD).

The nebulizing gas flow was 3 L/min, drying gas flow 10 L/min, and interface, desolvation line, and heating block temperatures were set at 300, 250, and 400 °C, respectively.

#### 4.2.2. Total Saponin Content

Total saponin content was determined using the spectrophotometric method described by Le et al. [32]. The crude extract was re-dissolved in ethanol and adjusted to a final volume of 10 mL. The solution was filtered through a 0.45 µm PTFE syringe filter (Sigma, Steinheim, Germany) prior to analysis. A stock solution (1.5 mg/mL) was prepared and subsequently diluted to obtain concentrations of 500, 250, 125, 62.5, and 31.25 µg/mL. An aliquot (0.25 mL) of each solution was transferred into test tubes and evaporated to dryness in a water bath at 65 °C. After cooling to room temperature, 0.25 mL of 8% (*w*/*v*) vanillin solution in ethanol was added, followed by 2.5 mL of 72% (*v*/*v*) sulfuric acid added carefully. The reaction mixture was vortexed and incubated in a water bath at 60 °C for 15 min, then cooled in an ice bath to stop the reaction. Absorbance was measured at 560 nm against a reagent blank using a UV–Vis spectrophotometer (Specord 40, Analytik Jena GmbH, Jena, Germany). Aescin was used as the reference standard, and the results were expressed as mg aescin equivalents per g of dry extract (mg AE/g).

#### 4.2.3. Total Phenolic Compounds

Total phenolic compounds were determined according to Sobatinasab et al. [33]. The Folin–Ciocalteu method was applied for the evaluation of TPC [34]. The absorbance was assessed at 765 nm, and TPC was expressed as tannic acid equivalent per g of dry weight (DW) of the sample (TAEg—1DW).

#### 4.2.4. Non-Volatile Analysis

Derivatization and analysis were performed according to the procedure described by Pachura et al. [35]. The crude ethanolic extract of *S. gigantea* (5 mg) was placed in a GC-MS vial, and next, 500 µL of pyridine and 50 µL of the derivatizing reagent N,O-bis(trimethylsilyl)trifluoroacetamide (BSTFA, Sigma-Aldrich) were added to the samples. Resveratrol was added as an internal standard. The analyses were performed in triplicate. The derivatization process was carried out on a heating plate at 70 °C for 20 min. Analysis of *S. gigantea* extract was performed on a Shimadzu GCMS QP 2020 (Shimadzu, Kyoto, Japan) equipped with a Zebron ZB-5 MSi column (30 m × 0.25 mm × 0.25 µm; Phenomenex, Torrance, CA, USA). The following program was applied: initial temperature was 180 °C and held for 1 min, then the temperature was increased by 3 °C·min^−1^ to 300 °C and held for 35 min. Scanning (2 scan·s^−1^) was performed with a collection of mass between 40 and 800 *m*/*z* in electronic impact (EI) mode at 70 eV. Samples were injected at a 1:50 split ratio, and helium gas was used as the carrier gas at a flow rate of 0.95 mL·min^−1^. 

#### 4.2.5. Volatiles Analysis

Volatiles presented in crude ethanolic extract of *S. gigantea* were assessed by hydrodistillation using the Deryng apparatus (100 mg of extract) (WPL, Gliwice, Poland). Hydrodistillation was applied to the crude ethanolic extract to recover volatile and semi-volatile constituents remaining in the extract matrix after solvent extraction. This approach does not reflect the native essential oil composition of the plant material but allows complementary characterization of extract-associated volatiles. The volatiles were collected in cyclohexane (Sigma-Aldrich, Steinheim, Germany) containing the 2-undecanone as an internal standard [34]. Hydrodistillation was carried out for 1 h since steam was condensed turn back in the process. EOs fractions, collected in cyclohexane, were centrifuged, dried under anhydrous sodium sulfate, kept in a vial, and stored at −27 °C until GC-MS analysis was performed. The analyses were performed in triplicate. The composition of volatile compounds was analyzed using a Shimadzu GCMS-QP2020 system (Shimadzu, Kyoto, Japan). Separation of compounds was achieved on a Zebron ZB-5MSi capillary column (30 m × 0.25 mm × 0.25 µm; Phenomenex, Torrance, CA, USA). GC-MS analysis parameters were as follows: scan range of 40–450 *m*/*z* with a speed of 1 scans·s^−1^. The carrier gas used was helium with a column flow of 0.93 mL·min^−1^, with a split ratio of 1:49. The oven temperature program was as follows: 50 °C as initial temperature to 250 °C at a rate of 3 °C·min^−1^, and kept for 3 min. Total time of analysis was 69.67 min. The injection volume was 1 µL at 260 °C. The quantification of identified *C. gigantea* constituents was carried out by peak area normalization against the added internal standard. Identification of volatile compounds was performed on the basis of the approach published by Pachura et al. [35]. In brief, the following methods were used to identify the components of the EOs: (a) a comparison of the obtained mass spectra with the databases NIST 23 (National Institute of Standards and Technology) and FFNSC (the mass spectra of flavors and fragrances of natural and synthetic compounds); (b) a comparison of the calculated linear retention indices (LRIs) using a retention indices calculator with values presented in NIST 23 National Institute of Standards and Technology; NIST/EPA/NIH Mass Spectral Library (NIST 23); Gaithersburg, MD, USA and FFNSC [36]; Mass spectra of flavors and fragrances of natural and synthetic compounds (3rd ed.); Wiley. Excel macro provided by Lucero et al. [37] was applied for this calculation; (c) a comparison of the retention times of the unknown compounds with authentic standards. Two software packages were used for the analysis: AMDIS (v. 2.73) and the ACD/Spectrus Processor v. 2025 (Advanced Chemistry Development, Toronto, ON, Canada).

### 4.3. Antimicrobial Assay and Biological Activity

#### 4.3.1. Test Microorganisms

The obtained extracts of *S. gigantea* were tested against a panel of microorganisms, including Gram-positive cocci: *Staphylococcus aureus* PCM 2054 (ATCC 25923) and PCM 458, *Staphylococcus pseudintermedius* PCM 2791, *Staphylococcus epidermidis* PCM 2118 (ATCC 14990), *Enterococcus faecalis* ATCC 29212 and *Enterococcus hirae* PCM 2559 (ATCC 10541); Gram-positive endospore-forming rods: *Bacillus cereus* PCM 2019 (ATCC 11778); Gram-negative rods: *Escherichia coli* PCM 2057 (ATCC 25922), *Pseudomonas aeruginosa* PCM 2058, *Salmonella enterica* subsp. *enterica* serotype Gallinarum PCM 2658; methicillin resistance strains: *S. aureus* MRSA strain ATCC 3144 and MRSA ATCC 33592, *S. epidermidis* MRSE PCM 2532; yeast *Candida albicans* PCM 2566, *Candida krusei* PCM F117, *Candida parapsilosis* Cp1. These strains came from the Polish Collection of Microorganisms (PCM, Institute of Immunology and Experimental Therapy, Polish Academy of Sciences, Wroclaw, Poland). Where indicated, original strain designations from the American Type Culture Collection (ATCC, Manassas, VA, USA) are provided. Bacterial cultures were maintained on TSA (Tryptic Soy agar, VWR, Leuven, Belgium), while yeast was on SDA (Sabouraud agar, VWR, Belgium) and stored on slants at 4 °C.

#### 4.3.2. Agar Disk Diffusion Assay

The antibacterial activity of *Solidago gigantea* extracts was evaluated using the agar disk diffusion method as previously described [15,38]. Briefly, standardized microbial suspensions (0.5 McFarland) were spread on Mueller–Hinton agar for bacteria, or Sabouraud agar for yeast. Sterile paper disks were loaded with the tested extracts (500 µg/disk) and placed on the agar surface. Gentamicin and amphotericin B were used as positive control, while 10% DMSO served as the negative control. Plates were incubated under appropriate conditions, and inhibition zones were measured. Experiments were performed in triplicate, and results were expressed as mean ± standard deviation (SD). Zone diameters were expressed in millimeters.

#### 4.3.3. Determination of Minimum Inhibitory Concentration (MICs) and Minimum Bactericidal Concentration (MBCs)

The minimum inhibitory concentrations (MIC_50_ and MIC_90_) were determined by a broth microdilution method in 96-well plates as previously described [15,39] with minor modifications. Standardized microbial suspensions (0.5 McFarland) were prepared and diluted to the appropriate inoculum density. The final inoculum density was approximately 5 × 10^5^ CFU/mL for bacteria, and 5 × 10^4^ CFU/mL for yeast.

The tested extract was dissolved in DMSO with Tween 80 (final concentrations ≤ 1% and ≤0.05%, respectively) and serially diluted in the range of 10 to 0.625 mg/mL. Appropriate controls, including growth, solvent, and sterility controls, were included.

After incubation under appropriate conditions, microbial growth was assessed spectrophotometrically. MIC was defined as the lowest concentration inhibiting visible growth. MIC_50_ and MIC_90_ were determined as the concentration of extract that inhibited microbial growth by 50% or ≥90%, respectively, compared to the growth control.

MBC/MFC (Minimum Bactericidal/Fungicidal Concentration) were determined by subculturing aliquots from wells without visible growth onto MHA or SDA (for bacteria or yeast) agar. The lowest concentration showing no colony growth was defined as the MBC/MFC.

#### 4.3.4. Cell Lines and Culture Conditions

The following cell lines were applied: Chinese hamster lung fibroblasts (V79, ECACC, catalog 93010723), normal human dermal fibroblasts (NHDF, LONZA, Catalog: CC-2511), human breast adenocarcinoma cells (MCF-7, ECACC, catalog No. 86012803), doxorubicin-resistant breast cancer cells (MCF-7/DX), human colorectal adenocarcinoma cells (LoVo, ECACC, catalog No. 87060101), doxorubicin-resistant colorectal cancer cells (LoVo/DX), and human monocytic leukemia cells (THP-1, ECACC, catalog 88081201). The collected cells used in this assay were obtained from the European Collection of Authenticated Cell Cultures (ECACC, UK Health Security Agency, Porton Down, Salisbury, UK) or LONZA (Verviers, Belgium).

V79, MCF-7, and MCF-7/DX were maintained in Eagle’s Minimum Essential Medium (EMEM) (Sartorius AG, Goettingen, Germany) supplemented with 10% fetal bovine serum (Capricorn Scientific GmbH, Ebsdorfergrund, Germany), 100 U/mL penicillin, and 100 µg/mL streptomycin (Capricorn Scientific GmbH, Ebsdorfergrund, Germany). LoVo, and LoVo/DX cells were grown in Dulbecco’s modified Eagle medium and Ham’s Nutrient Mixture F-12 (DMEM/Hams F-12) (Capricorn Scientific GmbH, Ebsdorfergrund, Germany) supplemented as before.

NHDF cells were cultured in DMEM supplemented with 10% FBS and antibiotics as described above. THP-1 cells were maintained in RPMI-1640 medium (Capricorn Scientific GmbH, Ebsdorfergrund, Germany) supplemented with 10% FBS and antibiotics.

Doxorubicin-resistant sublines (MCF-7/DX and LoVo/DX) were routinely cultured in the presence of low concentrations of doxorubicin (0.1 µg/mL) to maintain the resistant phenotype. This selection pressure is known to induce and stabilize P-glycoprotein (P-gp) overexpression. Cells were cultured in drug-free medium for at least 72 hated incubated at 37 °C in a humidified atmosphere containing 5% CO_2_ prior to experiments.

#### 4.3.5. Viability Assay

Cell viability was assessed using the MTT assay according to previously published protocols with minor modifications [8]. Cells were seeded into 96-well plates at a density of 1 × 10^4^ cells per well and allowed to adhere overnight. Cells were then treated with *Solidago gigantea* extract in concentrations ranging from 12.5 to 5000 µg/mL of extract for 24 h.

After incubation, MTT (Sigma, Steinheim, Germany) solution (1 mg/mL in RPMI1640 *w*/*o* phenol red, Capricorn Scientific GmbH, Ebsdorfergrund, Germany) was added to each well, and plates were incubated for an additional 2 h at 37 °C. The resulting formazan crystals were dissolved in 100 µL isopropanol, and absorbance was measured at 570 nm using a microplate reader (Multiscan Go, Thermo Scientific, Waltham, MA, USA). Half-maximal inhibitory concentration (IC_50_) values were calculated using nonlinear regression based on a four-parameter logistic (4PL) model with a variable Hill slope. IC_50_ values are presented as mean ± standard deviation (SD) calculated from five independent biological replicates.

To assess the selectivity of the cytotoxic effect, the selectivity index (SI) was calculated as the ratio of the IC_50_ value determined for normal human dermal fibroblasts (NHDF) to the IC_50_ value obtained for each cancer cell line.

#### 4.3.6. Sulforhodamine B (SRB) Assay

Cell proliferation was evaluated using the SRB assay as described previously [40]. After treatment, cells were fixed with cold trichloroacetic acid (TCA, 10% *w*/*v*, Sigma, Steinheim, Germany) and incubated at 4 °C for 1 h. Plates were washed with distilled water and stained with 0.4% SRB (Sigma, Steinheim, Germany) solution in 1% acetic acid for 30 min at room temperature.

Unbound dye was removed by washing with 1% acetic acid, and protein-bound SRB was dissolved in 10 mM Tris (Sigma, Steinheim, Germany) base solution (pH 10.5). Absorbance was measured at 540 nm using a microplate reader (Multiscan Go, Thermo Scientific). Based on seven absorbance measurements, including the signal recorded at time zero, untreated control growth, and drug-treated cell growth at five concentration levels, percentage growth was determined for each tested concentration. For drug concentrations at which the absorbance exceeded or equaled the time-zero value, growth was calculated relative to control cells, whereas for concentrations resulting in absorbance values below the time-zero level, growth was calculated relative to the initial measurement.

#### 4.3.7. Intracellular ROS Measurement

Intracellular reactive oxygen species (ROS) generation was measured using the DCF-DA assay (Sigma, Steinheim, Germany) according to established protocols [40]. Cells were seeded into black 96-well plates and treated with *S. gigantea* extract for the indicated time. After treatment, cells were incubated with 25 µM DCF-DA in serum-free medium for 30 min at 37 °C in the dark. The cells were dissolved in 20 mM Tris–HCl (pH 7.7) (Sigma, Steinheim, Germany) containing 0.2% sodium dodecyl sulfate (SDS, Sigma, Steinheim, Germany) to lyse and release the intracellular fluorescent substrate.

Fluorescence intensity was measured using a microplate reader at excitation/emission wavelengths of 485/535 nm using the Synergy HT microplate reader (BioTek Instruments, Winooski, VT, USA). ROS levels were expressed as a percentage relative to untreated control cells.

#### 4.3.8. Rhodamine 123 Accumulation Assay

The intracellular accumulation of rhodamine 123 (Sigma, Steinheim, Germany) was used to evaluate the influence of the tested compounds on P-glycoprotein–mediated transport. Cells were seeded in 96-well plates at a density of 10,000 cells per well and pretreated with the investigated compounds for 24 h. Following this incubation period, rhodamine 123 was added to each well to reach a final concentration of 12.5 μM, and cells were further incubated to allow intracellular uptake of the fluorescent probe.

After incubation, the plates were centrifuged at 500× *g* for 10 min, and the supernatant was carefully removed. Cell lysis was achieved by adding 20 mM Tris–HCl buffer (pH 7.7) supplemented with 0.2% sodium dodecyl sulfate (SDS), enabling the release of intracellular rhodamine 123. Fluorescence intensity was subsequently measured at an excitation wavelength of 485 nm and an emission wavelength of 535 nm using a Synergy HT microplate reader.

### 4.4. Statistical Analysis

All results in the tables are presented as mean ± SD (standard deviation) relative to the control. Statistical methods used were two-way ANOVA analysis of variance and Tukey’s post hoc test. The point of significance was established * *p* < 0.05. Statistical analyses were performed with Statistica v.13 software.

## 5. Conclusions

The results of this study demonstrate that *Solidago gigantea* extract exhibits complex, multi-target biological activity that is closely related to its diverse phytochemical composition. The extract is characterized by the coexistence of polyphenolic, lipophilic, and volatile fractions, which together contribute to its broad spectrum of biological effects.

The high content of phenolic compounds, particularly chlorogenic acid and flavonoids such as rutin and quercetin derivatives, is reflected in pronounced antioxidant and cytoprotective properties, including effective modulation of intracellular reactive oxygen species (ROS). At the same time, the extract showed selective antiproliferative and cytotoxic activity toward cancer cells, while exerting only limited effects on normal cells, suggesting its potential role in chemoprevention.

In addition, the extract demonstrated significant antibacterial activity against Gram-positive bacteria, including methicillin-resistant strains, highlighting its potential in the control of opportunistic pathogens. The observed biological effects are likely the result of synergistic interactions between multiple classes of compounds, including polyphenols, triterpenoids, and volatile terpenes.

Importantly, this study provides an integrated link between multi-fraction phytochemical composition and mechanistic biological effects, including oxidative stress modulation and multidrug resistance-related processes, which have been rarely addressed simultaneously in previous studies on *S. gigantea*.

Overall, *S. gigantea*, as an invasive and widely available plant species, represents a promising source of bioactive compounds with potential applications in chemoprevention, antimicrobial strategies, and adjunctive cancer therapy. These findings highlight the potential of combining phytochemical profiling with functional assays as an effective approach for identifying multi-target plant-derived agents. Further studies are warranted to elucidate the underlying molecular mechanisms and to evaluate their efficacy in in vivo models.

## Figures and Tables

**Table 1:** *S. gigantea* ethanolic extract phenolic acids and flavonoids content (mg/g).

Chemical Group	Compound	Content (mg/g)
Phenolic acids	Chlorogenic acid	23.57 ± 1.75
	Ferulic acid	13.28 ± 0.56
	Protocatechuic acid	3.04 ± 0.11
	Caffeic acid	1.64 ± 0.09
	Syringic acid	1.17 ± 0.10
	*p*-Coumaric acid	7.06 ± 0.34
	Vanillic acid	Tr
Flavonoids	Rutin (Quercetin-3-O-rutinoside)	19.47 ± 1.01
	Quercitrin (Quercetin-3-O-rhamnoside)	14.80 ± 0.88
	Quercetin	2.07 ± 0.23
	Neohesperidin	0.86 ± 0.11
	Kaempferol	0.34 ± 0.09
	Taxifolin (Dihydroquercetin)	Tr
	Polydatin (Resveratrol-3-O-glucoside)	Tr
Phenolic alcohol	Coniferyl alcohol	Tr

Tr—trace (below limit of quantification, LOQ); Values are mean ± standard deviation (SD).

**Table 2:** GC-MS table of derivatized fraction of *Solidago gigantea* extract.

KI	Peak Name	KI Exp.	KI Lit.	Content (mg/g)
1	Palmitic Acid	2045	2049	6.39 ± 0.45
2	Phytol	2169	2171	1.31 ± 0.10
3	Linoleic acid	2207	2210	4.15 ± 0.31
4	α-Linolenic acid	2212	2218	3.60 ± 0.27
5	Stearic acid	2245	2243	1.12 ± 0.09
6	8,15-Labdanediol	2409	2417	0.30 ± 0.02
7	Dimorphecolic acid	2432	2431	0.22 ± 0.02
8	Arachidic acid	2446	2443	0.59 ± 0.05
9	Variabilin	2511	2512	1.93 ± 0.14
10	Heptacosane	2701	2700	3.38 ± 0.25
11	Variabilin isomer	2533	2526	5.78 ± 0.42
12	*n*-Nonacosane	2899	2900	19.18 ± 1.34
13	1-Hexacosanol	2935	2940	16.81 ± 1.18
14	*n*-Triacontane	3001	3000	2.38 ± 0.18
15	*n*-Hentriacontane	3100	3100	21.37 ± 1.50
16	*n*-Dotriacontane	3200	3200	1.03 ± 0.08
17	Octacosanoic acid	3229	3233	1.27 ± 0.15
18	Triacontanol	3249	3251	0.87 ± 0.07
19	Stigmasterol	3290	3286	0.93 ± 0.09
20	*n*-Tritriacontane	3298	3300	4.11 ± 0.67
21	1-Triacontanol	3302	3306	10.58 ± 0.88
22	β-Amyrin	3365	3368	2.97 ± 0.19
23	Tritriacontane, 3-methyl-	3379	3376	1.04 ± 0.08
24	α-Amyrin	3401	3406	1.70 ± 0.13
25	Triacontanoic acid	3422	3419	1.81 ± 0.14
26	Olean-12-en-3β-ol acetate	3441	3438	1.41 ± 0.11
27	Germanicol acetate	3478	3480	1.24 ± 0.09
28	Erythrodiol	3504	3501	15.55 ± 1.06
29	Uvaol	3545	3540	9.01 ± 0.55
30	Lup-20(29)-ene, 3,28-bis[oxy]-, (3β)-	3561	3558	2.98 ± 0.21
31	Ursolic aldehyde	3675	3669	0.56 ± 0.07

KI exp.—retention index calculated according to *n*-alkanes; KI lit.—retention index calculated according to NIST23. Values are expressed as mean ± SD (*n* = 3). Quantification was performed using an internal standard (semi-quantitative analysis).

**Table 3:** GC-MS profile of VOCs in extract.

No.	Name	KI Exp.	KI Lit.	%
1	Hexanal	800	800	6.03 ± 0.74
2	*trans*-2-Hexenal	848	850	0.72 ± 0.11
3	α-Pinene	931	933	9.12 ± 0.89
4	Camphene	948	953	0.29 ± 0.04
5	Thuja-2,4(10)-diene	952	953	0.20 ± 0.03
6	Benzaldehyde	961	960	0.61 ± 0.09
7	Sabinene	971	972	1.10 ± 0.17
8	β-Pinene	976	978	2.78 ± 0.39
9	Myrcene	988	991	1.33 ± 0.20
10	Limonene	1028	1030	1.15 ± 0.18
11	Eucalyptol	1031	1032	0.45 ± 0.07
12	Benzeneacetaldehyde	1042	1045	0.30 ± 0.05
13	γ-Terpinene	1056	1058	0.23 ± 0.03
14	Sabinene hydrate	1069	1069	0.29 ± 0.04
15	α-Terpineol	1084	1087	1.05 ± 0.16
16	Linalool	1100	1101	0.92 ± 0.14
17	α-Campholenal	1124	1125	2.95 ± 0.44
18	*trans*-Pinocarveol	1139	1140	6.68 ± 0.88
19	*trans*-Verbenol	1144	1155	0.76 ± 0.11
20	Pinocarvone	1159	1162	0.02 ± 0.01
21	Isoborneol	1169	1165	0.96 ± 0.14
22	Myrtenal	1193	1197	2.10 ± 0.24
23	Verbenone	1205	1028	2.12 ± 0.21
24	Bornyl acetate	1282	1285	1.54 ± 0.19
25	β-Cubebene	1386	1382	1.89 ± 0.13
26	*trans*-β-Caryophyllene	1418	1423	1.25 ± 0.19
27	α-Humulene	1454	1454	1.58 ± 0.12
28	Germacrene D	1479	1480	0.82 ± 0.14
29	*epi*-Cubebol	1493	1498	0.74 ± 0.11
30	γ-Cadinene	1513	1512	0.59 ± 0.09
31	Germacrene B	1553	1557	0.90 ± 0.11
32	Caryophyllene oxide II	1565	1567	0.89 ± 0.13
33	Spathulenol	1574	1576	9.69 ± 1.35
34	Caryophyllene oxide	1579	1587	15.68 ± 2.07
35	Salvial-4(14)-en-1-one	1590	1596	1.72 ± 0.20
36	Humulene epoxide II	1607	1613	14.19 ± 1.87
37	Allo-Aromandendrene epoxide	1644	1644	1.63 ± 0.19
38	Germacra-4(15),5,10(14)-trien-1-alpha-ol	1687	1683	4.74 ± 0.24

KI exp.—retention index calculated according to *n*-alkanes; KI lit.—retention index calculated according to NIST23. Values are mean ± standard deviation (SD).

**Table 4:** Inhibition zones (mm) produced by *Solidago gigantea* extract in the agar diffusion assay, expressed as percentages of the positive control (values in parentheses).

Strain	*S. gigantea* Extract mm (%)	Positive Control mm
*Staphylococcus aureus* PCM 2054	13.0 ± 0.33 (48.8) ^c,d^	26.7 ± 0.5 ^a^
*Staphylococcus aureus* PCM 458	10.7 ± 0.38 (39.5) ^d^	27.0 ± 0.33 ^a^
*Staphylococcus aureus* MRSA ATCC 33592	11.7 ± 0.19 (100.0) ^d^	11.7 ± 0.19 ^d^
*Staphylococcus aureus* MRSA ATCC 3144	12.3 ± 0.19 (100.0) ^c,d^	12.0 ± 0.33 ^d^
*Staphylococcus epidermidis* PCM 2118	22.4 ± 0.33 (70.4) ^b^	27.0 ± 0.33 ^a^
*Staphylococcus epidermidis* MRSE PCM 2532	15.0 ± 0.33 (100.0) ^c,d^	15.0 ± 0.33 ^cd^
*Staphylococcus pseudintermedius* PCM 2791	11.3 ± 0.19 (39.5) ^d^	28.7 ± 0.19 ^a^
*Enterococcus hirae* PCM 2559	11.3 ± 0.19 (50.7) ^d^	22.3 ± 0.19 ^b^
*Escherichia coli* PCM 2057	1.3 ± 0.19 (4.8) *	28.0 ± 0.33 *
*Candida albicans* PCM 2566	0.0 ± 0.00 (0.0) *	20.7 ± 0.51 *
*Candida krusei* F117	0.0 ± 0.00 (0.0) *	22.5 ± 0.42 *
*Candida parapsilosis* Cp1	0.0 ± 0.00 (0.0) *	21.2 ± 0.31 *

Values are presented as mean ± standard deviation (SD, *n* = 3). Values in parentheses represent inhibition zones expressed as percentages of the positive control (gentamicin for bacteria and amphotericin B for yeasts). Different letters within each column indicate statistically significant differences between strains (Tukey HSD test, *p* < 0.05). *—not subjected to statistical analysis.

**Table 5:** Bacteriostatic (MIC_50_ and MIC_90_) and bactericidal/fungicidal (MBC/MFC) activity of *Solidago gigantea* extract (mg/mL) against tested pathogens.

Tested Strains	MIC_50_	MIC_90_	MBC/MFC
		mg/mL	
Gram-positive bacterial strains			
*Staphylococcus aureus* PCM 2054	0.9 ± 0.1 ^a^	3.9 ± 0.1 ^a^	2.5
*Staphylococcus aureus* PCM MRSA 3144	3.7 ± 0.5 ^b^	8.9 ± 0.7 ^b^	5
*Staphylococcus epidermidis* PCM 2118	3.6 ± 0.1 ^b^	8.1 ± 0.1 ^b^	5
*Staphylococcus epidermidis* PCM MRSE 2532	4.5 ± 0.1 ^bc^	8.3 ± 0.2 ^b^	5
*Staphylococcus pseudintermedius* PCM 2791	1.7 ± 0.1 ^a^	4.4 ± 0.1 ^a^	5
*Enterococcus faecalis* PCM 29212	5.1 ± 0.1 ^c^	8.4 ± 0.1 ^b^	10R
*Enterococcus hirae* PCM 2559	4.1 ± 0.1 ^b^	9.6 ± 0.3 ^b^	10R
*Bacillus cereus* PCM 2019	2.0 ± 0.1 ^a^	4.6 ± 0.3 ^a^	5
Gram-negative bacterial strains			
*Escherichia coli* PCM 2057	>10	>10	>10
*Pseudomonas aeruginosa* PCM 2058	>10	>10	>10
*Salmonella* Gallinarum PCM 2658	>10	>10	>10
Yeast			
*Candida albicans* PCM 2566	>10	>10	>10
*Candida krusei* F117	>10	>10	>10
*Candida parapsilosis* Cp1 (38)	>10	>10	>10

MIC_50_ and MIC_90_—Minimum Inhibitory Concentration of 50% and 90% of cells, respectively; MBC/MFC—Minimum Bactericidal/Fungicidal Concentration; > no bacteriostatic or bactericidal effect within the range of doses tested; ± standard deviation (SD); R—cell growth reduction; the values in columns marked with the same letters are not significantly different (Tukey HSD, *p* < 0.05).

**Table 6:** Cytotoxic activity of *Solidago gigantea* extract determined by the MTT assay.

Cell Line	IC_50_ (µg/mL)	Sensitivity Profile
MCF-7	78.2 ± 9.4	High sensitivity
MCF-7/DX	362.1 ± 38.5	Reduced sensitivity (cross-resistance)
A549	274.6 ± 31.7	Moderate sensitivity
LoVo	98.9 ± 11.2	High sensitivity
LoVo/DX	50.7 ± 7.8	Very high sensitivity
THP-1	1862.3 ± 210.5	Low sensitivity
NHDF	~7180 ± 580	Very low toxicity

Note: ± standard deviation (SD); IC_50_ values were calculated using a four-parameter logistic model based on five biological replicates. For NHDF cells, the IC_50_ value was estimated by extrapolation beyond the tested concentration range.

**Table 7:** Selectivity index (SI) of *Solidago gigantea* extract toward cancer cells. SI was calculated as the ratio of IC_50_ determined for NHDF cells to the IC_50_ value obtained for each cancer cell line.

Cell Line	SI
MCF-7	92.0
MCF-7/DX	19.8
A549	26.2
LoVo	72.7
LoVo/DX	142.0
THP-1	3.9

Higher SI values indicate greater selectivity of cytotoxic action toward cancer cells relative to normal fibroblasts.

**Table 8:** MRM transitions used for LC-MS/MS analysis.

Compound	Ionization Mode	Precursor Ion [*m*/*z*]	Product Ions [*m*/*z*]
Chlorogenic acid	negative	353.00	191.3, 85.0, 93.05
Ferulic acid	negative	193.40	163.05, 134.95, 178.95
Protocatechuic acid	negative	153.40	109.00, 108.00, 80.90
Caffeic acid	negative	179.40	135.05, 133.95, 107.00
Syringic acid	negative	197.40	181.85, 122.90, 86.95
*p*-Coumaric acid	negative	165.10	100.90, 68.75, 132.95
Vanillic acid	negative	167.40	152.05, 122.90, 107.90
Rutin	negative	609.30	300.15, 301.10, 275.25
Quercitrin	negative	447.00	301.00, 255.00, 271.00
Quercetin	negative	301.20	151.00, 179.00, 121.00
Neohesperidin	positive	611.10	303.00, 165.90, 125.00
Kaempferol	negative	285.00	93.15, 117.10, 185.20
Taxifolin	negative	303.00	285.00, 125.00, 151.00
Polydatin	negative	389.00	321.00, 343.10
Coniferyl alcohol	negative	179.40	88.90, 58.90, 70.90

## Data Availability

The original contributions presented in this study are included in the article and Supplementary Materials. Further inquiries can be directed to the corresponding authors.

## Supplementary Materials

The following supporting information can be downloaded at: https://www.mdpi.com/article/10.3390/molecules31101552/s1, including GC-MS chromatograms of TMS-derivatized and volatile fractions.

## Abbreviations

The following abbreviations are used in this manuscript:

AE/g	Aescin equivalents per gram
AMR	Antimicrobial resistance
BSTFA	N,O-bis(trimethylsilyl)trifluoroacetamide
CFU	Colony forming units
DCF-DA	2′,7′-dichlorodihydrofluorescein diacetate
DMSO	Dimethyl sulfoxide
GC–MS	Gas chromatography–mass spectrometry
IC_50_	Half-maximal inhibitory concentration
KI (RI)	Kovats retention index
LC–MS/MS	Liquid chromatography–tandem mass spectrometry
LOQ	Limit of quantification
MBC	Minimum Bactericidal Concentration
MDR	Multidrug resistance
MIC_50_	Minimum Inhibitory Concentration for 50% of isolates
MIC_90_	Minimum Inhibitory Concentration for 90% of isolates
MRM	Multiple reaction monitoring
MRSA	Methicillin-resistant *Staphylococcus aureus*
MRSE	Methicillin-resistant *Staphylococcus epidermidis*
MTT	3-(4,5-dimethylthiazol-2-yl)-2,5-diphenyltetrazolium bromide assay
NHDF	Normal human dermal fibroblasts
P-gp	P-glycoprotein
ROS	Reactive oxygen species
SI	Selectivity index
SRB	Sulforhodamine B assay
TMS	Trimethylsilyl
VOC(s)	Volatile organic compounds
