# Supplementary material for: Invasive Giant Goldenrod (Solidago gigantea Aiton): Phytochemical Profiling and Evaluation of Chemopreventive and Antimicrobial Activities
Source: Molecules. 2026 May 7;31(10):1552. doi: 10.3390/molecules31101552 (PMC13209318; doi:10.3390/molecules31101552)

Figure S1.

GC-MS chromatogram of volatile fraction in investigated *S. gigantea* extract processed in MestreNova

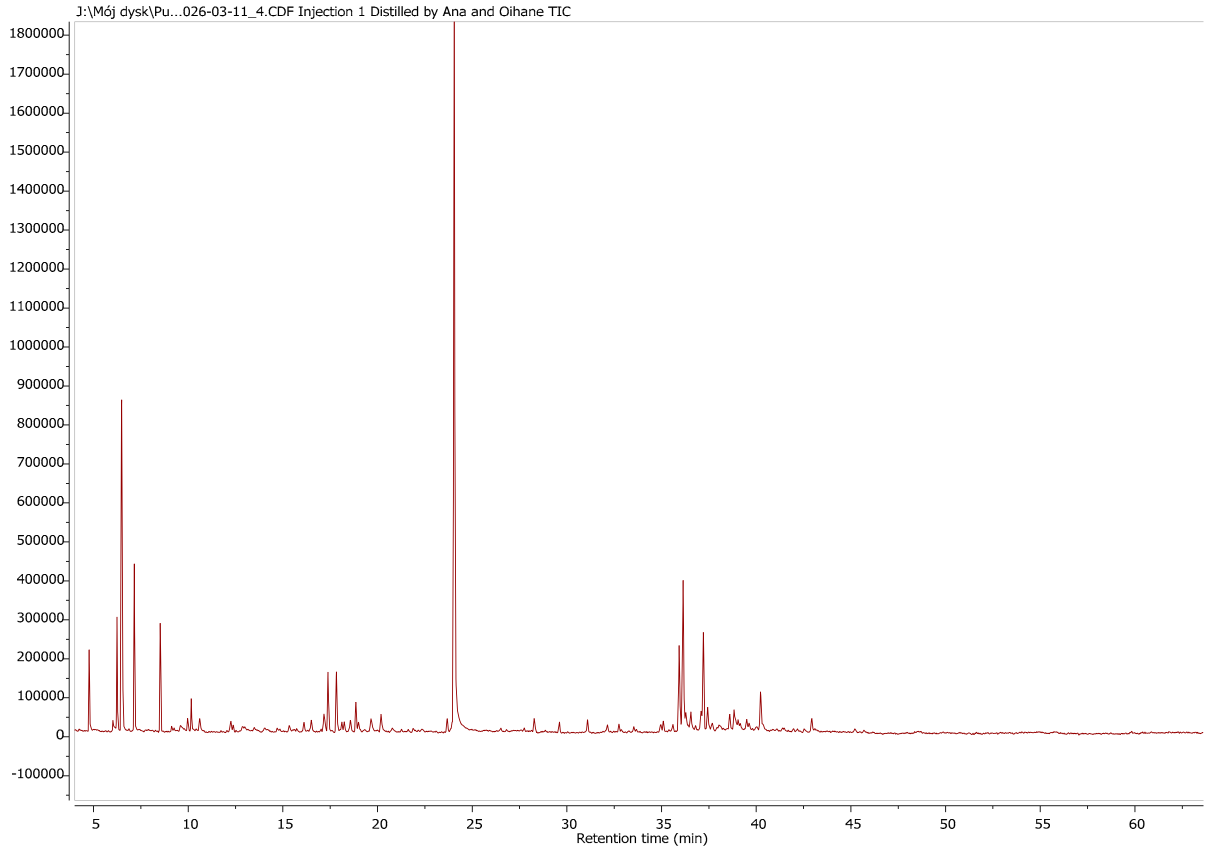

Figure S2.

GC-MS chromatogram of BSTFSA derivatized fraction of *S. gigantea* extract processes in Spectrus Processor

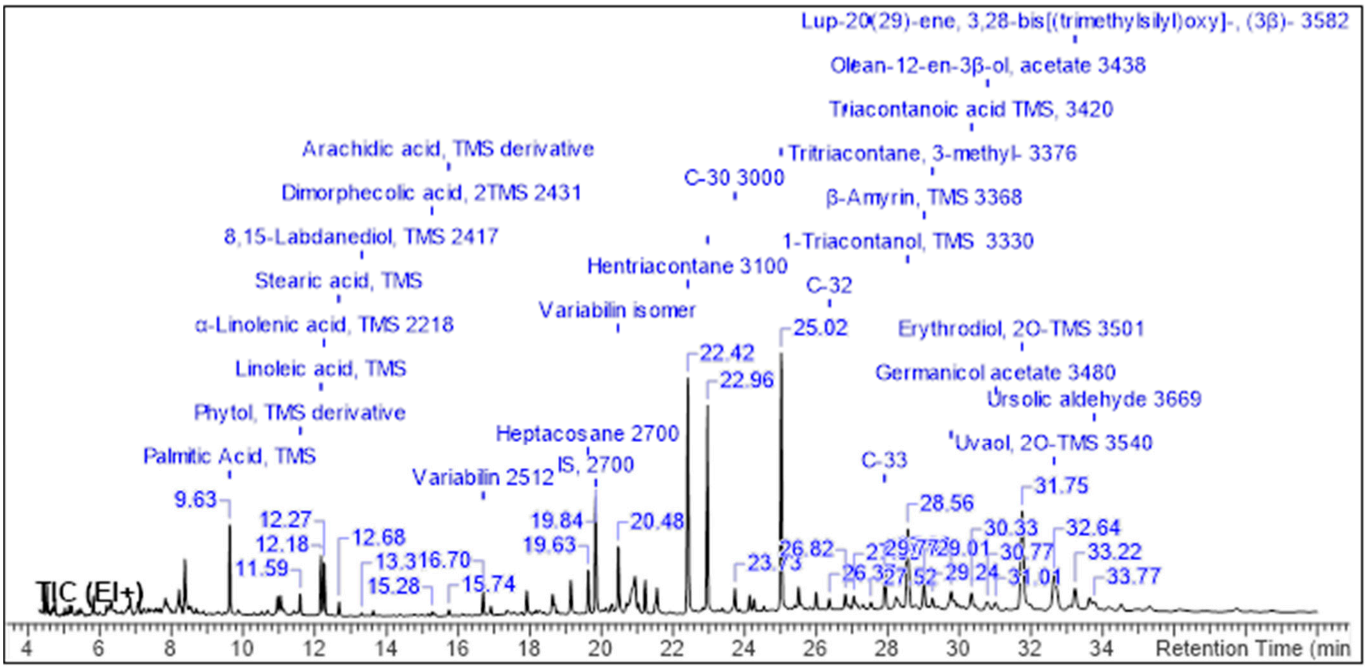

Supplement: Supplementary file 1 [file molecules-31-01552-s001.zip › molecules-4260319-supplementary.pdf]
